# Exploring the Immunodominant Epitopes of SARS-CoV-2 Nucleocapsid Protein as Exposure Biomarker

**DOI:** 10.7759/cureus.34827

**Published:** 2023-02-10

**Authors:** Kapil Vashisht, Bharti Goyal, Rahul Pasupureddy, Byoung-Kuk Na, Ho-Joon Shin, Dibakar Sahu, Sajal De, Soumyananda Chakraborti, Kailash C Pandey

**Affiliations:** 1 Parasite-Host Biology, Indian Council of Medical Research-National Institute of Malaria Research (ICMR-NIMR), Delhi, IND; 2 Biological Sciences, Academy of Scientific and Innovative Research (AcSIR), Ghaziabad, IND; 3 Parasitology and Institute of Health Sciences, Gyeongsang National University College of Medicine, Jinju, KOR; 4 Tropical Infectious Disease Cooperation Laboraory, Ajou University School of Medicine, Suwon, KOR; 5 Pulmonology Department, All India Institute of Medical Sciences, Raipur, IND

**Keywords:** immunodominant epitopes, protein microarray, nucleocapsid protein, covid-19, sars-cov-2

## Abstract

Background

The nucleocapsid protein (N protein) of SARS-CoV-2 is undeniably a potent target for the development of diagnostic tools due to its abundant expression and lower immune evasion pressure compared to spike (S) protein.

Methods

Blood samples of active COVID-19 infections (n=71) and post-COVID-19 (n=11) were collected from a tertiary care hospital in India; pre-COVID-19 (n=12) sera samples served as controls. Real-time reverse transcriptase-PCR (rRT-PCR) confirmed pooled sera samples (n=5) were used with PEPperCHIP® SARS-CoV-2 Proteome Microarray (PEPperPRINT GmbH, Germany) to screen immunodominant epitopes of SARS-CoV-2. Highly immunodominant epitopes were then commercially synthesized and further validated for their immunoreactivity by dot-blot and ELISA.

Results

The lowest detectable concentration (LDC) of the N1 peptide in the dot-blot assay was 12.5 µg demonstrating it to be fairly immunoreactive compared to control sera. IgG titers against the contiguous peptide (N2: 156AIVLQLPQGTTLPKGFYAEGS176) was found to be significantly higher (p=0.018) in post-COVID-19 compared to pre-COVID-19 control sera. These results suggested that N2-specific IgG titers buildup over time as expected in post-COVID-19 sera samples, while a non-significant immunoreactivity of the N2 peptide was also observed in active-COVID-19 sera samples. However, there were no significant differences in the total IgG titers between active COVID-19 infections, post-COVID-19 and pre-COVID-19 controls.

Conclusion

The N2-specific IgG titers in post-COVID-19 samples demonstrated the potential of N protein as an exposure biomarker, particularly in sero-surveillance studies.

## Introduction

The pandemic coronavirus disease-2019 (COVID-19) caused by Severe Acute Respiratory Syndrome Coronavirus 2 (SARS-CoV-2) wreaked unprecedented havoc, with over six million deaths globally. The pathology of COVID-19 ranges from asymptomatic/moderate to severe infections, often resulting in fatal outcomes with pneumonia and acute respiratory distress. The gold standard diagnostic tool for the detection of COVID-19 is real-time reverse transcriptase-PCR (rRT-PCR), which can detect the presence of nucleoprotein (N), envelope (E), RNA-dependent RNA polymerase (RdRp) and open reading frame 1ab (ORF 1ab) genes in nasopharyngeal swabs of suspected individuals [1]. Amongst the antigen-based tests, N and E proteins serve as two main target antigens, while the presence of antibodies IgM, IgG, and IgA could be detected within a week’s interval of initial infection [2]. SARS-CoV-2 is highly contagious and has rapidly evolved into ~25 clades so far, and the number is growing continuously. Based on data (genomes sequences) available on Global Initiative on Sharing Avian Influenza Database (www.gisaid.org), all sequences were classified into several clades and subclades such as S, O, L, V, G, GH, GR, GV and GRY. In fact, subclades like GR, GH, and GV have evolved from the G clade only. Interestingly several variants of concerns (VOCs) like alpha (B.1.1.7), beta (B.1.351), gamma (P.1), and delta (B.1.617.2) also evolved from G clade. Analysis of millions of SARS-CoV-2 genomes also revealed that the evolutionary (mutation) rate of the SARS-CoV-2 genome is approximately 1.1×10-3 substitutions/site/year, which is lower compared to other RNA viruses [3]. The most prominent variant of the current times, Omicron, was observed to be phylogenetically unique compared to all other SARS-CoV-2 variants, producing a monophyletic clade [4]. India has already suffered three major COVID-19 waves; however, the evolving nature of SARS-CoV-2 variants poses a constant threat of future outbreaks. SARS-CoV-2 possesses a single-stranded positive-sense RNA virus encoding four structural proteins: spike (S), envelope (E), membrane (M), and nucleocapsid (N) proteins. In the viral proteome, multifunctional nucleocapsid (N) protein is the most abundantly expressed protein, with approximately 1000 copies of N protein incorporated in each virion, constituting a major component of the capsid [5]. Globally, the N protein of SARS-CoV-2 has been implicated in multiple studies for its diagnostic potential [6-10]. Lesser genomic mutations (SNPs: single nucleotide polymorphisms) have been observed in N protein compared to spike (S) protein, suggesting lower immune-evasion pressure [11]. Therefore, immunodominant peptide epitopes of N protein are attractive targets for serological diagnosis of COVID-19. Protein microarray has critical implications in the identification of immunoreactive epitopes from different antigens of various parasites, such as Plasmodium, in a cost-effective and robust manner [12]. Given the lack of robust serological markers of SARS-CoV-2, we sought to identify highly immunoreactive epitopes through a peptide microarray using SARS-CoV-2 positive sera samples from India.

## Materials and methods

The study was approved by the Institutional Ethics Committees (IECs), AIIMS, Raipur [1379/IEC-AIIMSRPR/2020] & ICMR-NIMR, Delhi [PHB/NIMR/EC/2020/145]. Single time-point blood samples were collected from a tertiary care hospital (All India Institute of Medical Sciences, Raipur, India) from December 2020 to January 2021 after taking informed consent from study subjects. RT-PCR-confirmed SARS-CoV-2 infected patients (n=82) were recruited for the study and classified as active COVID-19 and post-COVID-19 (rRT-PCR negative after COVID-19 detection) (Table 1). Blood samples collected before the COVID-19 pandemic (n=12) were taken as controls. The blood samples were collected at different time points within 0-4 weeks intervals from the date of detection of COVID-19 positive. To explore the highly immunoreactive epitopes of the SARS-CoV-2, the PEPperCHIP® SARS-CoV-2 Proteome Microarray (PEPperPRINT GmbH, Germany) was procured. The microarray slide contained all SARS-CoV-2 protein sequences (ORF1a/b, S protein, ORF3a, Envelope protein, Membrane glycoprotein, ORF6, ORF7a, ORF8, N protein, and ORF10) in the form of overlapping peptides (4,883) of 13 amino acid length. Hemagglutinin (HA) and polio peptides were imprinted on the periphery of the microarray slide as control spots.

**Table 1:** Classification of the COVID-19 infected patients included in the study Classification of COVID-19-infected patient samples based on clinical characteristics. Mild patients required light oxygen support; moderate patients required high oxygen support; severe patients required ventilator support.

Active COVID-19 (71)	Post-COVID-19 (11)
Mild (29)	Mild (3)
Moderate (31)	Moderate (3)
Severe (11)	Severe (5)

The microarray experiment was performed as per the manufacturer’s instructions [12]. Briefly, the microarray slide was incubated in PBS containing 0.05% Tween 20 (PBS-T, pH 7.4) for 15 minutes and further incubated with Rockland Blocking Buffer (RL) (Rockland Immunochemicals) for 30 minutes at room temperature (RT). Pooled sera from SARS-CoV-2 infected patients (n=5) diluted (1:100) in 10% RL/PBS-T was used for the incubation of microarray slide on an orbital shaker at 4˚C overnight. The slide was washed thrice with PBS-T for 1 minute each (washing step). Goat anti-human IgG (Fc) tagged with DyLight 550 (Invitrogen, USA) antibodies at 1:2500 dilution were incubated onto the microarray slide for 45 minutes, and the washing step was repeated. For staining HA and polio control peptide spots, an anti-HA antibody (1:2000) tagged with Cy5 was incubated for 45 minutes; the wash step was repeated and dried with the air stream. The microarray slide was scanned using a microarray scanner (Molecular Devices, USA) at Translational Health Sciences and Technology Institute (THSTI), Faridabad, Haryana, with a resolution of 20 mm, and fluorescent read-outs of DyLight 550 and Cy5 dye were recorded. The Pep-Slide analyzer software algorithm calculated the mean foreground intensities (background-deducted intensities) of the spots in duplicates.

Highly immunoreactive peptides identified from the PEPperCHIP® SARS-CoV-2 Proteome Microarray (PEPperPRINT GmbH, Germany) were commercially synthesized. For preliminary validation, dot-blot assays were conducted to assess the immunoreactivity of peptide N1, commercially procured from Genetix Biotech Asia Pvt. Ltd. India. Briefly, 50 µg of the serially diluted peptide was used to coat methanol activated and PBS-equilibrated PVDF membrane using Bio-Rad dot-blot SF apparatus and dried using a vacuum pump. The membrane was incubated with the pooled sera of SARS-CoV-2 infected patients (n=5) and blocked with 5% skimmed milk at 4˚C overnight. After washing with PBST, the PVDF strip was incubated with rabbit anti-human IgG secondary antibody tagged with horseradish peroxidase (HRP) (diluted 1:2500) (Invitrogen, USA) for 1 hour at room temperature before developing with DAB (3,3'-Diaminobenzidine tetrahydrochloride) substrate.

Direct-Enzyme-Linked Immunosorbent Assay (ELISA) was performed to estimate the total IgG titers and IgG specific to the contiguous peptide N2, commercially procured from Peptron, South Korea. To quantitatively estimate the peptide N2-specific IgG titers, 50 µg peptide N2 was coated on a 96-well ELISA plate, incubated overnight at 4˚C and further blocked with blocking buffer (3% BSA in 1X PBS) for 1 hr. SARS-CoV-2 infected and pre-COVID-19 sera samples (1:100) were incubated for 1 hour at room temperature. To estimate the total IgG titers in SARS-CoV-2 infected serum samples and pre-COVID-19 controls, goat anti-human IgG (Fc specific) antibodies (Sigma-Aldrich, USA) at 1:5000 dilution were coated on a 96-well ELISA plate overnight at 4˚C. After washing with 1X PBS, the plate was incubated with SARS-CoV-2 infected serum samples and pre COVID-19 controls (diluted 1:100) for 2 hours. The plates were washed with wash buffer (0.05% Tween-20 in 1XPBS) and further incubated with rabbit anti-human IgG secondary antibody tagged with HRP (Invitrogen, USA) with a dilution of 1:5000 in 1X PBS buffer for 1 hour. The plate was developed by adding 100 µl of 3,3',5,5'-Tetramethylbenzidine (TMB) substrate; the reaction was stopped using 0.2 N H2SO4 after 20 minutes, and absorbance was recorded in a microplate reader at 450 nm. Multiple sequence alignments (MSA) of the peptide N2 of SARS-CoV-2 with respective sections of the HKU1, MERS, OC43 and SARS-CoV were generated using ClustalW software using default parameters.

## Results

Proteome microarray

**Figure 1:**
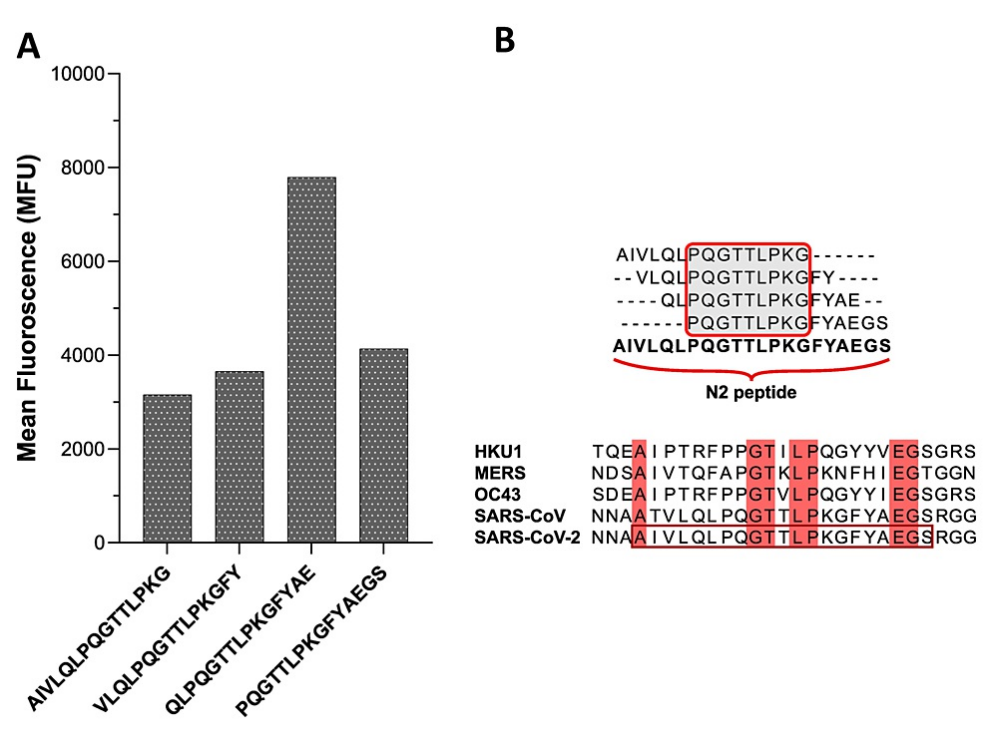
Immunodominant peptide-epitope of Nucleocapsid (N) protein of SARS-CoV-2 Immunodominant peptide-epitope of Nucleocapsid (N) protein of SARS-CoV-2. A. Bar-graph showing the reactivity of different peptide epitopes. B. Consensus sequence from the most immunodominant epitopes resulting in the N2 peptide and MSA of the peptide N2 region with other coronaviruses (HKU1, MERS, OC43, SARS-CoV).

The PEPperCHIP® SARS-CoV-2 proteome microarray contained all the structural and non-structural proteins in overlapping linear peptides in duplicate along with the HA and polio control peptides on the periphery of the microarray slide. Four immunoreactive epitopes of the N protein were identified from the mean foreground intensities of individual peptides indicated as bar graphs in Figure 1A. Peptide (N1: 160QLPQGTTLPKGFYAE174) was observed as the most interacting epitope, along with other contiguous peptides a consensus sequence (N2: 156AIVLQLPQGTTLPKGFYAEGS176) was generated (Figure 1B). Multiple sequence alignments of this region with other coronaviruses such as HKU1, MERS, OC43, SARS-CoV and the peptide N2 of SARS-CoV-2 revealed approximately 33% similarity (Figure 1B).

Dot blot and ELISA with immunodominant epitopes of N protein

**Figure 2:**
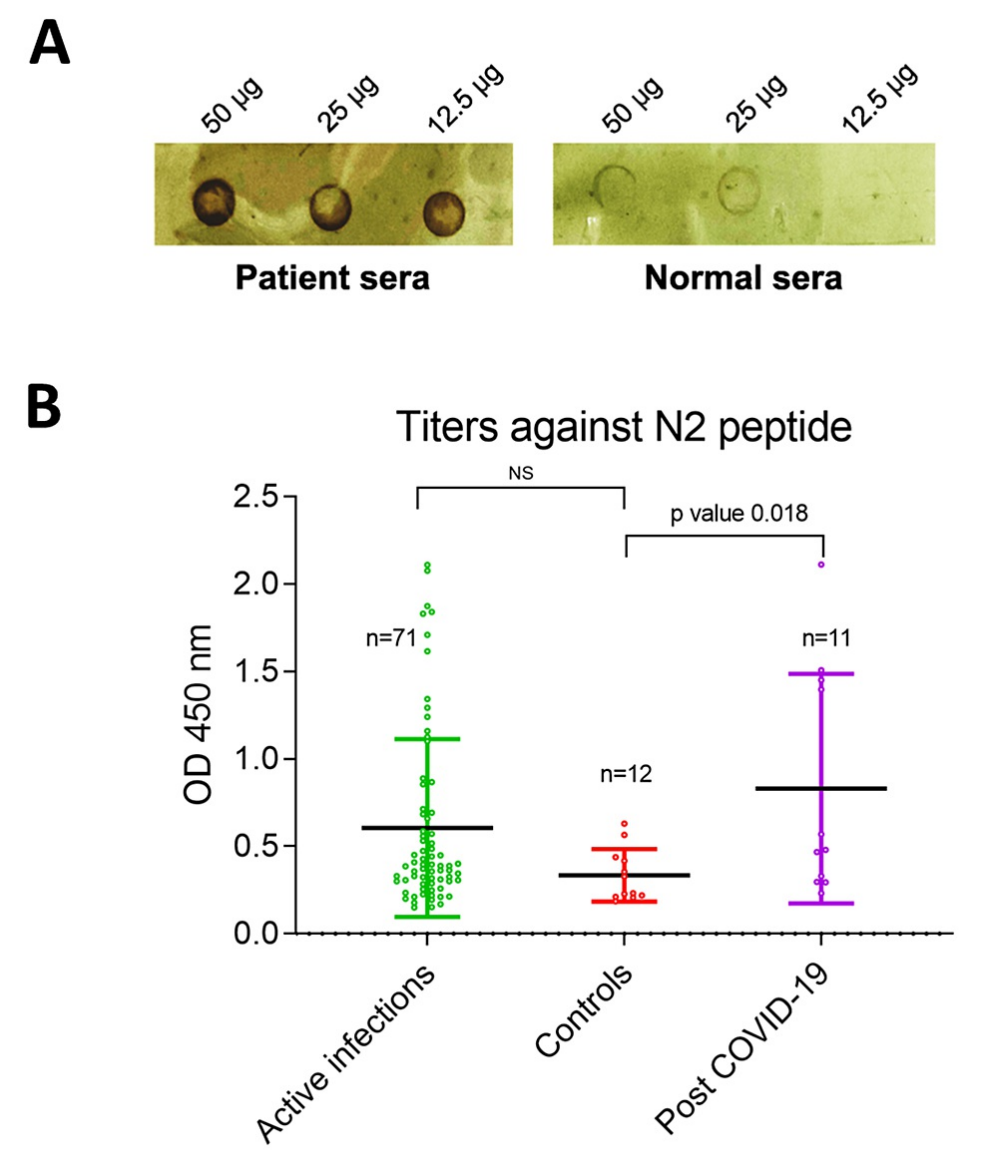
Dot blot and ELISA assays to analyze the immunoreactivity of N1 and N2 peptides A. Preliminary dot-blot immunoassay to qualitatively analyze the sensitivity of the immunodominant peptide epitope N1. B. ELISA depicting the immunoreactivity of N2 peptide; significantly segregating controls vs. post-COVID-19 subjects (p=0.018); non-significant in controls vs. active infections.

Peptide N1 was commercially synthesized to validate the immunoreactivity with the SARS-CoV-2 infected sera samples through dot blot assays, where the lowest detectable concentration of peptide N1; LDC_N1=12.5 µg was observed (Figure 2A). There was clear visual signal in the COVID-19 infected patient’s sera as compared to the healthy sera. To assess IgG tiers further quantitatively IgG tiers against the contiguous peptide N2, ELISA was performed. The N2 peptide was tested with a bigger pool of SARS-CoV-2 positive samples (n=82) and pre-COVID-19 controls (n=12) (Figure 2B). The immunoreactivity of the N2 peptide was mild in the most active COVID-19 infections (n=71), which can be attributed to lower IgG levels in the initial weeks of infection. Therefore, the difference in the peptide N2-specific IgG titers between the controls and active COVID-19 infections was found to be non-significant. Unsurprisingly, post-COVID-19 sera samples (n=11) depicted higher IgG titers, indicating a buildup of stronger immune responses, and a significant difference in the IgG titers was observed (p=0.018) as compared to the controls (Figure 2B).

**Figure 3:**
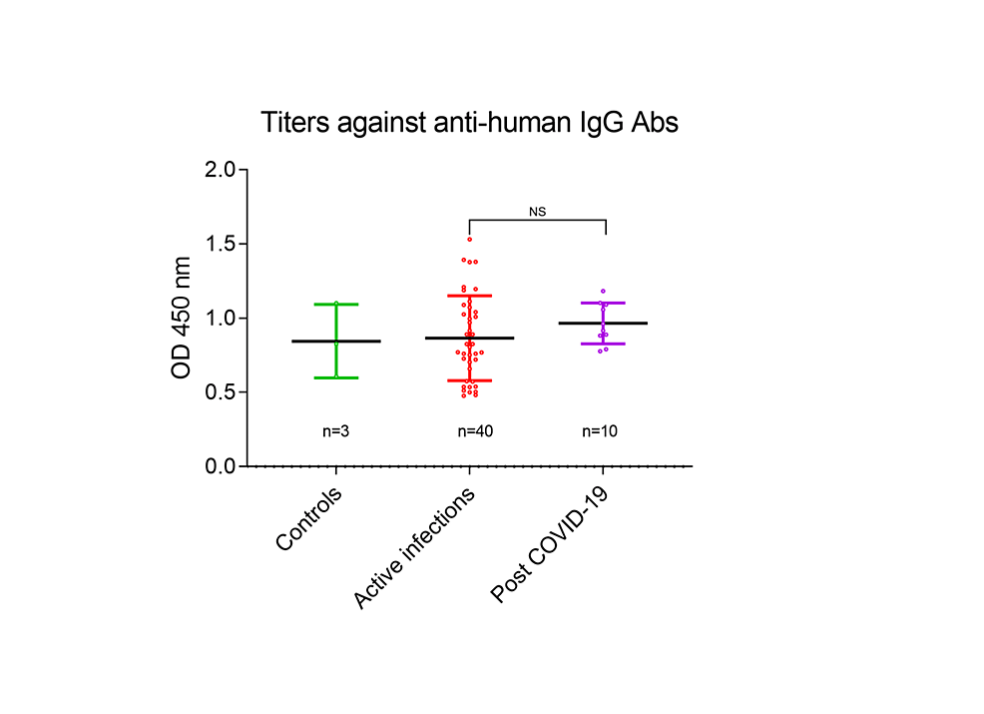
Total IgG titers in COVID-19 active infections, post-COVID-19 subjects, and controls ELISA depicting the total IgG titers in the sera samples with no significant difference between the groups (controls, active infections, and post-COVID-19).

It was important to assess the total IgG titers in active and post-COVID-19 infections to accurately estimate the peptide N2-specific IgG titers. We observed that there was no significant difference in the total IgG titers of the sera samples between controls (n=3), active (n=40), and post-COVID-19 (n=10) groups (Figure 3), confirming that IgG reactivity against the peptide N2 is specific.

**Figure 4:**
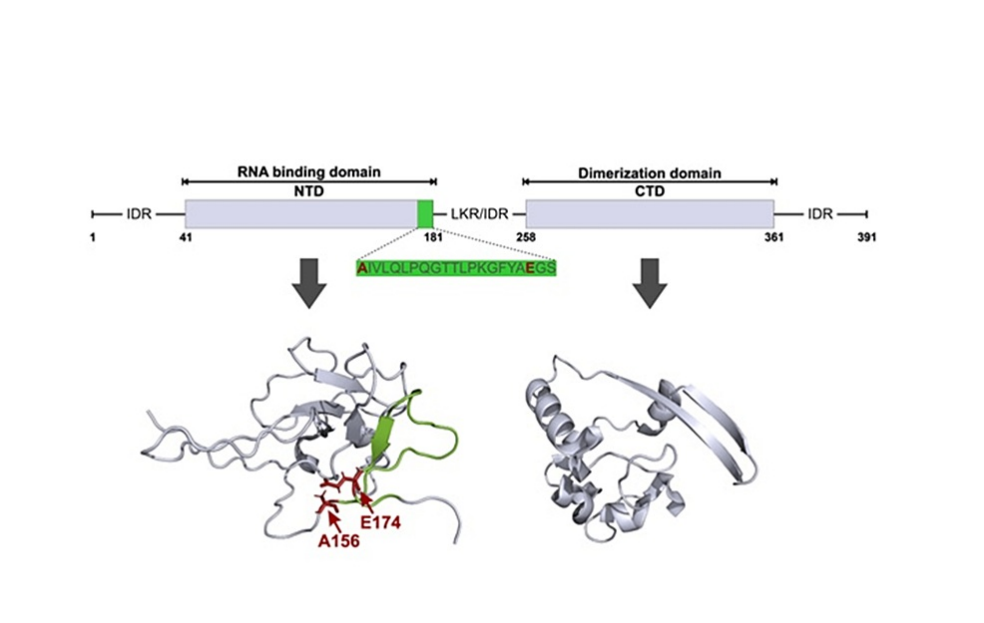
Domain structure of the SARS-CoV-2 (N) protein and location of the N2 peptide Schematic indicating the various domains of the SARS CoV-2 (N) protein. Structure of the N-terminal RNA binding domain (PDB ID: 6YI3) and dimerization domain (PDB ID: 6YUN) of SARS-CoV-2 N protein. The immunodominant epitope (green) and RNA binding residues (red) are highlighted.

The peptide N2 lies at the C-terminal of the RNA binding domain of the N protein, followed by a linker/intrinsically disordered region connecting the dimerization domain (Figure 4). The structures of the RNA binding and dimerization domains (PDB IDs- 6YI3 and 6YUN, respectively) are represented in Figure 4.

## Discussion

The present study exploited the protein microarray technology to identify immunoreactive epitopes from the SARS-CoV-2 peptides printed as linear peptides of 13 amino acid lengths. The four immunoreactive peptides with high foreground intensities were located on the same stretch and yielded a consensus sequence in the form of peptide N2. The peptide N2 reacted differently to the antibodies present in the SARS-CoV-2 sera samples classified as active and post-COVID-19 infections. This is important to note that in the active COVID-19 infections, the blood samples were collected within 0-4-week intervals, and hence the responses were not significant compared to controls. But in post-COVID-19 infections, the IgG responses against the peptide N2 were significant as compared to controls. However, the total IgG titers between pre-COVID-19 controls, active and post-COVID-19 groups were not significantly different. Thereby, our results validate that the N2 peptide can have implications in sero-surveillance studies at the community level. To the best of our knowledge, the present study is the first report of screening immunoreactive epitopes from Indian SARS-CoV-2 patients. The samples were collected during the second COVID-19 wave in India when the delta variant of SARS-CoV-2 was predominantly circulating. It would be interesting to investigate the immunoreactivity of peptide N2 with other variants of SARS-CoV-2 circulating in the populations. Interestingly, the N2 peptide residues identified in our study closely overlapped with peptide epitopes mentioned in previous studies on subjects from different geographies [8,9,13], thus, corroborating the diagnostic potential of N2 peptide in SARS-CoV-2 diagnoses.

Multiple sequence alignment (MSA) of sequences covering the highlighted epitopes revealed that the N2 peptide is highly conserved with SARS-CoV though it shared minimal sequence similarity with other human β-coronaviruses, suggesting minimal cross-reactivity (Figure 1B). Structurally, the N protein is comprised of three intrinsically disordered regions (IDRs): An N-terminal RNA binding domain (NTD) and a C-terminal oligomerization domain (CTD), which are linked by a central Ser/Arg-rich linker region (LKR) (Figure 4) [14]. We observed that the N2 peptide lies at the N- terminal RNA binding domain (Figure 4). Interestingly, a recent survey on the Indian SARS-CoV-2 variants reported a mutation A156S (first residue of the N2 peptide) [15], and its conservation across β-coronaviruses implicates its crucial role in RNA binding.

Contrary to the gold-standard real-time reverse transcriptase-polymerase chain reaction (RT-PCR) for SARS-CoV-2 detection, serological diagnosis is a much more convenient and less resource-intensive tool. Rapid diagnostic tests (RDTs) are usually based on full-length target proteins but have certain limitations, such as high production/logistic costs, stability, and cross-reactivity issues. Peptide-epitope-based RDTs can offer an excellent alternative, often depicting robust immunoreactivity matching/surpassing the full-length proteins. Hereby, we report an immunodominant peptide from SARS-CoV-2 N protein identified from Indian COVID-19-positive subjects. Thorough validation with larger cohorts and temporally segregated immune responses are needed to accelerate the translational potential of such peptide-epitope-based serological markers.

## Conclusions

The present study explored the immunodominant epitopes from the SARS-CoV-2 proteome and demonstrated that the peptide N2-specific IgG titers might be exploited as an exposure biomarker in sero-surveillance studies at the community level. The present study included COVID-19 subjects infected with the Delta variant of the SARS-CoV-2 during the second COVID-19 wave in India; however, it would be interesting to investigate the immunoreactivity of peptide N2 in the infections of other variants such as Omicron of the SARS-CoV-2 in prospective studies. Moreover, the subjects in the present study were unvaccinated; therefore, it would be worthwhile to investigate the immunoreactivity of the peptide N2 in vaccinated subjects also.
